# Intake of Grains and Dietary Fiber and Prostate Cancer Aggressiveness by Race

**DOI:** 10.1155/2012/323296

**Published:** 2012-11-13

**Authors:** Fred Tabung, Susan E. Steck, L. Joseph Su, James L. Mohler, Elizabeth T. H. Fontham, Jeannette T. Bensen, James R. Hebert, Hongmei Zhang, Lenore Arab

**Affiliations:** ^1^Department of Epidemiology and Biostatistics, Arnold School of Public Health, University of South Carolina, Columbia, SC 29208, USA; ^2^Cancer Prevention and Control Program, Arnold School of Public Health, University of South Carolina, Columbia, SC 29208, USA; ^3^Division of Cancer Control and Population Sciences, National Cancer Institute, Bethesda, MD 20892, USA; ^4^Roswell Park Cancer Institute, Buffalo, NY 14263, USA; ^5^School of Public Health, Louisiana State University Health Sciences Center, New Orleans, LA 70112, USA; ^6^Department of Epidemiology, University of North Carolina at Chapel Hill, Chapel Hill, NC 27599, USA; ^7^Lineberger Comprehensive Cancer Center, University of North Carolina at Chapel Hill, Chapel Hill, NC 27599, USA; ^8^David Geffen School of Medicine, University of California, Los Angeles, CA 90095, USA

## Abstract

*Purpose*. To examine the associations among intake of refined grains, whole grains and dietary fiber and aggressiveness of prostate cancer in African Americans (AA, *n* = 930) and European Americans (EA, *n* = 993) in a population-based, case-only study (The North Carolina-Louisiana Prostate Cancer Project, PCaP). *Methods*. Prostate cancer aggressiveness was categorized as high, intermediate or low based on Gleason grade, PSA level and clinical stage. Dietary intake was assessed utilizing the NCI Diet History Questionnaire. Logistic regression (comparing high to intermediate/low aggressive cancers) and polytomous regression with adjustment for potential confounders were used to determine odds of high prostate cancer aggressiveness with intake of refined grains, whole grains and dietary fiber from all sources. *Results*. An inverse association with aggressive prostate cancer was observed in the 2nd and 3rd tertiles of total fiber intake (OR = 0.70; 95% CI, 0.50–0.97 and OR = 0.61; 95% CI, 0.40–0.93, resp.) as compared to the lowest tertile of intake. In the race-stratified analyses, inverse associations were observed in the 3rd tertile of total fiber intake for EA (OR = 0.44; 95% CI, 0.23–0.87) and the 2nd tertile of intake for AA (OR = 0.57; 95% CI, 0.35–0.95). *Conclusions*. Dietary fiber intake was inversely associated with aggressive prostate cancer among both AA and EA men.

## 1. Introduction

In the United States, prostate cancer currently is the most frequently diagnosed cancer in men and the second leading cause of cancer death in men after lung cancer [1, 2]. Clinically, prostate cancer is diagnosed as local (confined to the prostate), regional, or advanced (distant spread) [3]. Risk factors for prostate cancer may differ by disease aggressiveness. Therefore, determinants of mortality also might differ from those of incidence. Results from studies of various suspected risk factors for prostate cancer aggressiveness, such as body mass index (BMI = weight  (kg)/height  (m)^2^) and smoking, have been conflicting especially in the prostate-specific antigen (PSA) era (i.e., the past 20 years) [4]. Studies relying solely on incidence may have limited applicability to identifying risk factors for prostate cancer mortality because of the high survival rate of PSA-detected cancers [2]. 

African Americans (AAs) experience greater incidence and increased burden from late-stage diagnosis, aggressive tumor biology and much higher mortality compared to European Americans (EAs) [5, 6]. After adjusting for socioeconomic status, year and age at diagnosis, AA were found to be at increased risk of being diagnosed with nonlocalized disease [7], and this difference is in proportion to the corresponding racial difference in mortality. One potential explanation for racial disparities observed in prostate cancer is that AA may be more intensely exposed to deleterious nutritional factors that increase the risk of more aggressive prostate cancer relative to EA or, conversely, have reduced exposure to those dietary factors that may be beneficial [8]. The high prevalence of “latent” prostate cancer compared with clinically significant disease suggests that dietary factors influencing the later stages of prostate cancer progression may be relevant to effective intervention [9]. Epidemiological studies suggest that diet is a key factor in the etiology of aggressive prostate cancer (including cross-national comparisons of prostate cancer mortality) [10]. In an investigation of the effect of high intake of whole grains and refined grains on prostate cancer progression in men diagnosed with low-grade prostate cancer, the authors concluded that whole grain and bran from rye resulted in significantly lower plasma PSA levels compared with a cellulose-supplemented refined wheat diet [11]. This report further emphasizes the importance of investigating the role of diet in the biological processes involved in the aggressiveness of prostate cancer [9].

Grains account for about 25% of food energy in the US but an estimated 95% of grains available for consumption are refined [12]. Grains are stripped of their bran layers and germ during the refining process, which depletes many biologically active substances, such as fiber, antioxidants, minerals, and phytoestrogens, demonstrated to have beneficial effects on carcinogenesis [12, 13]. Fiber has been implicated in prostate cancer etiology; fiber increases sex hormone-binding globulin [14, 15] and improves insulin sensitivity [16–18], both of which may decrease risk of aggressive prostate cancer. Increased intake of whole grains and fiber or decreased intake of refined grains also may be plausibly associated with decreased risk of aggressive prostate cancer.

The majority of studies have focused on the association between whole grain intake and risk of gastrointestinal cancers, with some showing inverse associations [19, 20]. The relationship between grain consumption, dietary fiber intake, and prostate cancer aggressiveness by race has received very little attention in epidemiological research. Some studies have examined the adverse effects of refined grains and several cancers at the same time, with no particular emphasis on prostate cancer. Very few studies have concentrated on dietary fiber and risk of prostate cancer [21–24], and even fewer have examined this relationship with respect to disease aggressiveness. Of the few studies reporting on fiber/whole grain consumption and prostate cancer, results have been conflicting due partly to heterogeneity in design or analysis, which include differences in case selection (hospital case series versus population-based registry), race proportions (usually small, but variable proportions of AA) and inconsistent adjustment for potential confounders [13, 20, 25–28]. 

The purpose of this study was to examine the association between dietary fiber, whole and refined grains, and high prostate cancer aggressiveness in a previously conducted, population-based study in North Carolina and Louisiana. Approximately half of the participants were AA, thus allowing for the examination of racial differences in associations. 

## 2. Materials and Methods

The current study utilized data from the North Carolina-Louisiana Prostate Cancer Project (PCaP), a large, population-based, case-only study of prostate cancer conducted in North Carolina and Louisiana. PCaP research methods have been described [29]. Briefly, residents of North Carolina and Louisiana study areas with a first diagnosis of histologically confirmed adenocarcinoma of the prostate were eligible to participate if they: (1) were between 40 and 79 years old at diagnosis; (2) could complete the study interview in English; (3) did not live in an institution (e.g., nursing home); (4) were not cognitively impaired or in a severely debilitated physical state; and (5) were not under the influence of alcohol, severely medicated or apparently psychotic at the time of interview. Eligible men self-identified as either African-American (AA)/Black or Caucasian/Caucasian-American/White (herein defined as European-American or EA) in response to the open-ended interview questions “What is your race?” Research protocols were approved by the institutional review boards at the University of North Carolina at Chapel Hill, Louisiana State University Health Sciences Center and the Department of Defense Prostate Cancer Research Program, and all research subjects signed an informed consent prior to study participation.

Questionnaire data were collected during an in-person interview conducted by a trained nurse. Dietary intake factors used to define the main exposures were from the modified National Cancer Institute (NCI) diet history questionnaire (DHQ), with categorization into tertiles based on the distribution among the low and intermediate aggressive cases (referent cases). Participants were asked to recall their usual diet for the year prior to diagnosis. Refined grain and whole grain consumption were measured in number of servings per day. Total dietary fiber was measured in grams per day and was composed of soluble and insoluble fibers [30, 31] which also were analyzed separately for effects. Men with unreasonable estimates for total energy intake (<500 or >6,000 kcals/day) were excluded from the analyses. 

All prostate cancer cases in this study had a histologically confirmed adenocarcinoma of the prostate. They were classified based on clinical Gleason (histopathological) grade and stage, and serum PSA level at diagnosis as follows:

- high aggressive: Gleason sum ≥ 8 OR PSA > 20 ng/mL OR Gleason sum ≥ 7 and stage T3-T4,
- low aggressive: Gleason sum < 7 and stage T1-T2 and PSA < 10 ng/mL, and
- intermediate aggressive: all other cases.

In logistic regression analyses, the referent group consisted of research subjects with low and intermediate aggressive prostate cancer, with the high-risk group being subjects with high aggressive prostate cancer. In a separate analysis for comparison, high and intermediate aggressive cases were combined and compared to low-aggressive cases. 

In separate analyses, a dichotomous outcome variable based on Gleason sum alone was utilized. This variable was defined as high aggressive if Gleason sum ≥ 8 or =7 with a pattern of (4 + 3), and as low aggressive if Gleason sum < 7 or =7 with a pattern of (3 + 4).

### 2.1. Statistical Analysis

After excluding men with missing outcome data or unreasonable energy intakes, the data from 930 AA and 993 EA men were analyzed using SAS version 9.2. Statistical significance was set at *α* = 0.05 (two-tailed). Descriptive analyses (means, frequencies, and percentages) of the outcome, main exposures (refined grains, whole grains, and dietary fiber) and potential effect modifiers/confounders stratified by race and state were performed. Chi-square tests and *t*-tests were used to assess the differences between the two races.

Unconditional logistic regression was utilized to examine associations between aggressive prostate cancer and intake of refined grains, whole grains and dietary fiber (total, insoluble, and soluble), stratifying by race. The standard multivariate approach was used to adjust for total energy intake. Main exposures were categorized into tertiles based on the distribution among the low and intermediate aggressive cases. Age-adjusted crude odds ratios and 95% confidence intervals and the odds ratios adjusted for confounders were calculated for all tertiles with the first tertile as the referent. Potential confounders included age (continuous), race (except when stratifying by race), total energy intake (continuous), alcohol intake (continuous), education level (graduate school/professional degree, some college education or college graduate, high school or vocational/technical school graduate, some high school education or less than 8th grade), smoking status (current, former, and never smokers), physical activity (metabolic equivalents/week), fruit intake (servings/day), vegetable intake (servings/day), use of nutritional supplements (1–4 days/week, more than 5 days/week, and no use), use of NSAIDs (yes/no), screening history of prostate cancer (whether study participants had at least one PSA or digital rectal exam in the 12 months before prostate cancer diagnosis), body mass index (underweight (<18.5 kg/m^2^), normal weight (18.5 to <25.0 kg/m^2^), overweight (25.0 to <30.0 kg/m^2^), and obese (≥30.0 kg/m^2^)), and family history of prostate cancer (yes/no). A test for trend was conducted whereby the median of each tertile of main exposure was assigned to each participant accordingly. This variable was entered into the model as a continuous variable, and its *P* value was interpreted as the *P*_trend_.

The main focus of this study was the evaluation of the racial disparity between AA and EA with respect to refined grain, whole grain, and dietary fiber intake and prostate cancer aggressiveness. Therefore models were evaluated both stratified by race and across the unstratified sample. Polytomous logistic regression was utilized to evaluate the associations between all three levels of prostate cancer aggressiveness and intakes of refined grains, whole grains, total dietary fiber, soluble and insoluble fiber to gain a clearer picture of the association between nutrient intake and different levels of prostate cancer aggressiveness. 

## 3. Results

There was no racial difference (*P* = 0.74) by mean age at diagnosis (62 years for AA versus 64 years for EA; see Table 1). A lower percentage of AA (32%) compared to EA (37%) were current-smokers. More AA had education of “less than 8th grade” (31%) compared to EA (10%). A lower percentage of AAs (78%) had at least one prostate cancer screening in the 12 months before the cancer was diagnosed, compared to EAs (89%). The mean intake of refined grains was similar in AA (5.1 servings per day) and EA (4.5 servings per day), and the average intake of total energy was higher in AA (2631 kcal per day) than in EA (2324 kcal per day) (see Table 1). The mean intake of whole grains was similar in the two racial groups (1.0 whole grain servings for AA versus 1.2 servings for EA, *P* = 0.46). The mean intake of total dietary fiber was similar in AA (22.6 g/day) compared to EA (21.7 g/day), *P* = 0.11.

Odds ratios from the race-stratified and unstratified analyses of refined grains and whole grains intake revealed no statistically significant associations with aggressive prostate cancer across any of the tertiles of intake. The race-stratified and unstratified analyses yielded statistically significant results for dietary fiber intake and prostate cancer aggressiveness in the fully adjusted models. Results are presented in Tables 2 and 3 and described as follows.

Odds ratios from the refined grain model (Table 2) showed no significant associations either in the age-adjusted or in the fully adjusted models. For all subjects and for the EA-stratified analyses, a borderline inverse association was seen in the age-adjusted model for the 3rd tertile of whole grain intake ((OR = 0.77; 95% CI, 0.58–1.04), (OR = 0.64; 95% CI, 0.41–1.01), resp.). This was attenuated in the fully adjusted model ((OR = 0.88; 95% CI, 0.63–1.22), (OR = 0.66; 95% CI, 0.40–1.09), resp.). In the AA race stratum, no significant associations were found for whole grain intake in either the age-adjusted or fully adjusted models. 

The total dietary fiber model (Table 2), yielded an inverse association in the fully adjusted model for the 2nd (OR = 0.70; 95% CI, 0.50–0.97) and 3rd (OR = 0.61; 95% CI, 0.40–0.93) tertiles of intake for both racial groups combined, *P*_trend_ = 0.02. In the race-stratified analyses, odds ratios were significant in both the age-adjusted and fully adjusted models in the 3rd tertile of total dietary fiber intake for EA and in the 2nd tertile of intake for AA. For insoluble fiber, odds ratios for the two race groups combined were significant for the 3rd tertile of intake (OR = 0.61; 95% CI, 0.41–0.92). In EA, higher intake (3rd tertile) of insoluble fiber was inversely associated with aggressive prostate cancer (OR = 0.43; 95% CI, 0.22–0.82) while in AA, only a borderline inverse association was observed for the 2nd tertile of intake (OR = 0.63; 95% CI, 0.39–1.02). For soluble fiber, there was an inverse association for the two race groups combined. Odds ratios for the 2nd and 3rd tertiles of soluble fiber intake were OR = 0.69; 95% CI, 0.50–0.97 and OR = 0.64; 95% CI, 0.41–0.99, respectively. In EA, higher intake (3rd tertile) of soluble fiber was inversely associated with aggressive prostate cancer (OR = 0.46; 95% CI, 0.23–0.93) in the fully adjusted model, while in AA, no association was observed. Inverse associations appeared to be stronger in EA and increased in magnitude (see Table 2 for magnitudes of association) with higher intake of fiber (*P*_trend_ = 0.02 for total fiber, 0.01 for insoluble fiber, and 0.03 for soluble fiber).

When high and intermediate aggressive cases were combined and compared to low aggressive cases (data not shown), results were not materially different from those presented for refined grains and whole grains. For intake of dietary fiber (total, insoluble, and soluble), inverse associations were observed for high intake (3rd tertile) and the protective associations were restricted to EA. 

In separate analyses (data not shown), a two-level aggressiveness variable was used as the outcome based on Gleason sum only (see methods for definition of this variable). Results from these analyses were similar to those reported in Table 2, with total fiber, insoluble fiber and soluble fiber showing inverse associations with aggressiveness, particularly among EAs. For example, the OR for high aggressive prostate cancer (defined as Gleason sum of 7 with pattern of 4 + 3 or higher) in the third tertile of total fiber intake was 0.53, 95% CI, 0.29, 0.97 for EAs and was 0.89, 95% CI, 0.52, 1.51 for AAs after adjustment of the same variables as noted in the footnote of Table 2. The OR for total fiber intake was 0.72, 95% CI, 0.49, 1.06 for all subjects combined.

Odd ratios from polytomous logistic regression models (fully adjusted) showed significant inverse associations between dietary fiber intake (total, insoluble, and soluble), comparing high aggressive with low aggressive cases for the 2nd and 3rd tertiles of intake, in both racial groups combined (Table 3). In the refined grains and whole grains models, there were no significant associations in either racial group using polytomous regression.

## 4. Discussion

The associations between fiber, whole grain and refined grain intake, and prostate cancer aggressiveness were examined in a large sample of AA and EA prostate cancer cases from North Carolina and Louisiana. Total fiber, insoluble fiber, and soluble fiber all showed statistically significant inverse associations with prostate cancer aggressiveness that persisted after adjustment for potential confounders in both racial groups. In the polytomous models, the significant inverse association between dietary fiber and prostate cancer aggressiveness was observed only in comparisons between highly aggressive and low aggressive cancer; no significant associations were observed in the comparisons of intermediate aggressive cancers with low aggressive cancers. No other significant associations were observed, which suggests that fiber intake may be protective against aggressive prostate cancer, while refined grains may not be substantially related to the aggressiveness of prostate cancer. The protective association increased in magnitude with higher intake of dietary fiber (*P*_trend_ = 0.02) and appears to be stronger in EA than AA.

To the best of our knowledge, this is the first study that directly addresses the relationship between refined grains, whole grains, and dietary fiber and prostate cancer aggressiveness within a study population including a large proportion of AAs. Many studies examined the association between whole grains or dietary fiber and prostate cancer incidence, rather than the outcomes of disease aggressiveness or mortality, and results were mixed [20, 22, 23, 28, 32]. A few ecological studies examined associations between cereal intake and prostate cancer mortality. In a cross-national comparison of predictive factors for prostate cancer mortality, energy from cereal food sources were found significantly inversely associated with prostate cancer mortality [10]. Two other ecological studies provide further support showing that cereal intake is more strongly related to reduced prostate cancer mortality than meat or milk consumption are associated with increased prostate cancer mortality [33, 34]. 

In contrast, and more recently, Nimptsch et al. analyzed data from the Health Professionals Followup Study and reported positive associations between dietary fiber and high-grade prostate cancer and between whole grain intake and prostate cancer. However, these associations disappeared, or were attenuated, after restricting the data to men whose prostate cancers were PSA detected [27]. In a case-control study, Lewis et al. reported inverse associations between fiber intake and prostate cancer risk, but a positive association between total grain intake and prostate cancer risk [28]. However, the definition of grains did not distinguish between whole grains and refined grains. Suzuki et al. found no statistically significant association between dietary fiber intake and risk of advanced-stage or high-grade prostate cancer in the European Prospective Investigation into Cancer and Nutrition (EPIC) study, a large prospective cohort study conducted in 10 European countries [22]. However, a significant inverse association for fiber from fruit and prostate cancer risk at age 65 years or older was observed.

 Both strengths and limitations of the study merit discussion. A major advantage of this study is that it was population-based and included a large number of AA (*n* = 930; about 50% of the sample), a population underrepresented in most studies of diet and prostate cancer despite their high rate of disease. As with any epidemiological study of diet and cancer, misclassification of the exposure and confounding are potential limitations. Misclassification of dietary intake could have influenced the results, both because of the measurement error associated with the use of the NCI DHQ and because of the time period on which it focused. Diet in the year prior to cancer diagnosis may not be the most relevant etiologic timeframe for assessing risk of prostate cancer aggressiveness. The NCI DHQ was administered rigorously by trained and certified study nurses and the median time between diagnosis and interview was approximately four months. If there was some degree of recall bias, then it most likely would have been nondifferential because recall would not likely depend on disease aggressiveness (i.e., on which case-referent status was based). Also, despite errors there are good indications that diet is relatively stable in adulthood [35, 36].

Confounding cannot be ruled out completely in any observational study. An advantage with PCaP is that detailed data on many factors were collected, which allows for careful consideration of potential confounding factors, though residual or uncontrolled confounding could have been introduced with the categorization of variables or through error in measuring exposures and covariates or both. Stronger effects were found in the fully adjusted models compared to the age-adjusted models for dietary fiber (total, soluble, insoluble) in the race stratified and race-unstratified analysis (Table 2). Finally, multiple comparisons were conducted such that we cannot completely rule out the role of chance in explaining any of the statistically significant findings.

As a case-only study, PCaP has relevant implications for cancer epidemiology. Incidence has typically been considered as a more relevant endpoint than mortality, but this may not be true for prostate cancer, a typically indolent disease whose natural history depends on disease aggressiveness. Given the increased incidence of indolent prostate cancer since the advent of population-based PSA screening [37–39], this is very important to keep in mind when interpreting results of studies conducted in the last twenty years, as we have recently shown [40]. Studies focusing primarily on incidence may miss important associations with aggressive prostate cancer and, subsequent mortality from prostate cancer. Indeed, the new U.S. Preventive Services Task Force recommendations underline the importance of discounting PSA-detected prostate cancer to a large extent, especially in older men in whom incidence may rise to close to 100% but who have about zero chance of dying of prostate cancer [41]. The present study presents evidence for a role of dietary fiber in prostate cancer aggressiveness. Dietary fiber encompasses a wide range of components from many sources, and it is likely that these vary in their protection against prostate cancer aggressiveness. Future studies aimed at identifying specific components and sources of dietary fiber that are most protective may be useful. Furthermore, future studies focused on dietary patterns or other specific nutrients may shed further light on racial disparities in prostate cancer mortality. 

## Figures and Tables

**Table 1:** Characteristics of prostate cancer cases by race.

Characteristic	African Americans		European Americans		Difference testing
	(*n* = 930)		(*n* = 993)		
	Mean	SD	Mean	SD	
Mean age (years)	61.9	7.8	64.1	7.8	0.7426
	n	%	n	%	

Site					0.6391
Louisiana	452	48.6	472	47.5	
North Carolina	478	51.4	521	52.5	
Prostate cancer aggressiveness^a^					<0.0001
Low	431	46.3	556	56.0	
Intermediate	313	33.7	287	28.9	
High	186	20.0	150	15.1	
Tumor stage					0.0845
T1	507	54.5	547	55.1	
T2	389	41.8	421	42.4	
T3-T4	16	1.7	14	1.4	
Missing	18	1.9	11	1.1	
Categorized sum of Gleason score					0.019
≥8 or 7 with (4 + 3) pattern	201	21.7	173	17.5	
<7 or 7 with (3 + 4) pattern	724	78.3	817	82.5	
Education level					<0.0001
Graduate school/prof.degree	64	6.9	216	21.7	
Some college/college grad	270	29.1	412	41.5	
High school grad/vo-tec	308	33.1	269	27.1	
<8th grade/some high school	287	30.9	96	9.7	
Smoking status					<0.0001
Never	184	19.8	94	9.4	
Former	453	48.7	532	53.6	
Current	293	31.5	367	37.0	
Level of physical activity^b^					<0.0001
Vigorous	412	44.3	560	56.4	
Moderate	265	28.5	263	26.5	
No/Light	247	26.6	169	17.0	
Missing	6	0.6	1	0.1	
Use of nutritional supplements					<0.0001
None	310	33.3	537	54.1	
1–4 days/week	52	5.6	62	6.2	
5+ days/week	568	61.1	394	39.7	
Use of NSAIDs					<0.0001
Yes	408	43.9	666	67.1	
No	513	55.1	325	32.7	
Missing	9	0.9	2	0.2	
Multivitamin supplementation					<0.0001
No	605	65.1	458	46.1	
Yes	325	34.9	535	53.9	
Family history (first degree relative affected)					0.1561
No	688	74.0	762	76.7	
Yes	242	26.0	231	23.3	
Screening history (PSA or DRE)					<0.0001
No	207	22.3	113	11.3	
Yes	723	77.7	880	88.7	
Body mass index (weight (kg)/height (m^2^))					0.001
Under weight (<18.0)	9	1.0	3	0.3	
Normal weight (18.0–<25.0)	180	19.6	154	15.6	
Over weight (25.0–<30.0)	357	38.9	461	46.7	
Obese (30.0–<40.0)	325	35.4	335	34.0	
Highly obese (≥40.0)	47	5.1	33	3.4	

^a^Prostate cancer aggressiveness is defined as the severity of the cancer at diagnosis based on combinations of the Gleason score, morphologic stage, and PSA as follows: high aggressive: Gleason sum ≥ 8 OR PSA > 20 ng/mL OR Gleason sum ≥ 7 and stage T3c-T4c; low aggressive: Gleason sum < 7 and stage T1c-T2c and PSA < 10 ng/mL; intermediate aggressive: all other cases.

^b^Physical activity categories defined by MET-hrs/week as follows: vigorous: >17MET-hrs/week; moderate: >6.5 to ≤17 MET-hrs/week; and no/light: 0 to ≤6.5 MET-hrs/week.

**Table 2:** Age-adjusted and fully adjusted odds ratios for prostate cancer aggressiveness in relation to refined grain, whole grain and dietary fiber intake stratified by race.

	African Americans					European Americans					All subjects				
Exposure tertile^c^	*n* (cases/controls)	OR^a^	95% CI^a^	OR^b^	95% CI^b^	*n* (cases/controls)	OR^a^	95% CI^a^	OR^b^	95% CI^b^	*n* (cases/controls)	OR^a^	95% CI^a^	OR^b^	95% CI^b^
Refined grain intake															

T1	65/256	1.00	referent	1.00	referent	51/283	1.00	referent	1.00	referent	116/539	1.00	referent	1.00	referent
T2	47/246	1.01	0.66–1.55	0.99	0.62–1.60	52/271	0.99	0.66–1.50	0.96	0.61–1.50	99/517	1.01	0.75–1.35	0.98	0.71–1.36
T3	74/241	1.23	0.84–1.80	0.95	0.54–1.66	47/285	0.87	0.56–1.36	0.61	0.33–1.14	121/526	1.12	0.84–1.49	0.78	0.52–1.17
P^(trend)^			0.26		0.85			0.55		0.19			0.40		0.21
P^(race interaction)^	0.43														

Whole grain intake															

T1	95/320	1.00	referent	1.00	referent	46/230	1.00	referent	1.00	referent	141/550	1.00	referent	1.00	referent
T2	45/229	0.93	0.63–1.37	0.97	0.64–1.47	60/287	1.07	0.72–1.63	1.13	0.72–1.78	105/516	0.96	0.73–1.28	1.06	0.78–1.43
T3	46/194	1.02	0.68–1.51	1.13	0.73–1.76	44/322	0.64	0.41–1.01	0.66	0.40–1.09	90/516	0.77	0.58–1.04	0.88	0.63–1.22
P^(trend)^			0.69		0.62			0.02		0.89			0.17		0.15
P^(race interaction)^	0.14														

Total fiber intake															

T1	87/289	1.00	referent	1.00	referent	52/258	1.00	referent	1.00	referent	139/547	1.00	referent	1.00	referent
T2	55/232	0.63	0.41–0.98	0.57	0.35–0.93	59/285	0.86	0.57–1.29	0.78	0.48–1.25	114/517	0.73	0.55–0.98	0.70	0.50–0.97
T3	44/222	1.01	0.76–1.59	0.77	0.44–1.34	39/296	0.66	0.42–1.04	0.44	0.23–0.87	83/518	0.91	0.68–1.20	0.61	0.40–0.93
P^(trend)^			0.48		0.43			0.07		0.02			0.55		0.02
P^(race interaction)^	0.11														

Insoluble fiber intake															

T1	85/287	1.00	referent	1.00	referent	51/247	1.00	referent	1.00	referent	136/534	1.00	referent	1.00	referent
T2	60/235	0.67	0.44–1.02	0.63	0.39–1.02	55/285	0.87	0.57–1.39	0.78	0.49–1.29	116/520	0.76	0.57–1.02	0.75	0.54–1.04
T3	41/221	1.10	0.76–1.60	0.80	0.47–1.37	44/307	0.62	0.39–0.97	0.43	0.22–0.82	85/528	0.86	0.66–1.16	0.61	0.41–0.92
P^(trend)^			0.52		0.46			0.04		0.01			0.39		0.02
P^(race interaction)^	0.13														

Soluble fiber intake															

T1	86/286	1.00	referent	1.00	referent	55/263	1.00	referent	1.00	referent	143/549	1.00	referent	1.00	referent
T2	44/221	0.82	0.54–1.23	0.75	0.47–1.19	54/295	0.71	0.47–1.06	0.62	0.38–1.00	98/516	0.76	0.57–1.01	0.69	0.50–0.97
T3	56/236	1.17	0.79–1.72	0.84	0.47–1.53	41/281	0.72	0.46–1.13	0.46	0.23–0.93	97/517	0.97	0.73–1.30	0.64	0.41–0.99
P^(trend)^			0.12		0.62			0.97		0.03			0.97		0.65
P^(race interaction)^	0.48														

^a^adjusted for age only.

^b^adjusted for age, race (in race-unstratified models), total energy intake,education level, smoking status, physical activity, use of nutritional supplements, use of NSAIDs, prostate cancer screening history, family history of prostate cancer and body mass index.

^c^Tertile ranges and units are as follows.

Refined grain (servings/d): T1 ≤ 3.50, 3.5 < T2 ≤ 5.28, T3 > 5.28.

Whole grain (servings/d): T1 ≤ 0.63, 0.63 < T2 ≤ 1.33, T3 > 1.33.

Total fiber (grams/d): T1 ≤ 16.94, 16.94 < T2 ≤ 24.83, T3 > 24.83.

Insoluble fiber (grams/d): T1 ≤ 10.9, 10.9 < T2 ≤ 16.2, T3 > 16.2.

Soluble fiber (grams/d): T1 ≤ 5.81, 5.81 < T2 ≤ 8.86, T3 > 8.86.

**Table 3:** Fully adjusted^a^ odds ratios for prostate cancer aggressiveness in relation to refined grain, whole grain, and dietary fiber intake from polytomous regression models, with and without stratification by race.

Exposure tertile^b^	African Americans		European Americans		All subjects	
	OR	95% CI	OR	95% CI	OR	95% CI
Refined grain intake						

Intermediate versus low aggressive						
T1	1.00	referent	1.00	referent	1.00	referent
T2	1.01	0.67–1.52	0.88	0.61–1.27	0.95	0.72–1.24
T3	1.43	0.88–2.33	0.73	0.45–1.17	1.02	0.73–1.43
High versus low aggressive						
T1	1.00	referent	1.00	referent	1.00	referent
T2	0.99	0.60–1.65	0.91	0.57–1.45	0.96	0.69–1.35
T3	1.11	0.61–2.02	0.54	0.28–1.03	0.79	0.52–1.21

Whole grain intake						

Intermediate versus low aggressive						
T1	1.00	referent	1.00	referent	1.00	referent
T2	0.93	0.65–1.35	1.23	0.84–1.80	1.07	0.83–1.39
T3	1.06	0.72–1.56	0.93	0.63–1.39	0.98	0.79–1.30
High versus low aggressive						
T1	1.00	referent	1.00	referent	1.00	referent
T2	0.94	0.60–1.47	1.22	0.76–1.96	1.09	0.79–1.50
T3	1.16	0.73–1.87	0.65	0.39–1.09	0.87	0.62–1.23

Total fiber intake						

Intermediate versus low aggressive						
T1	1.00	referent	1.00	referent	1.00	referent
T2	1.33	0.60–1.34	0.92	0.62–1.36	0.92	0.70–1.21
T3	1.92	0.77–2.05	0.74	0.44–1.23	0.97	0.69–1.37
High versus low aggressive						
T1	1.00	referent	1.00	referent	1.00	referent
T2	1.00	0.33–0.92	0.75	0.46–1.24	0.67	0.47–0.95
T3	1.08	0.47–1.56	0.39	0.19–0.79	0.60	0.39–0.93

Insoluble fiber intake						

Intermediate versus low aggressive						
T1	1.00	referent	1.00	referent	1.00	referent
T2	0.89	0.60–1.34	0.92	0.62–1.36	1.09	0.83–1.44
T3	1.26	0.77–2.05	0.74	0.44–1.23	0.99	0.71–1.39
High versus low aggressive						
T1	1.00	referent	1.00	referent	1.00	referent
T2	0.55	0.33–0.92	0.75	0.46–1.24	0.76	0.54–1.08
T3	0.86	0.47–1.56	0.39	0.20–0.79	0.61	0.39–0.93

Soluble fiber intake						

Intermediate versus low aggressive						
T1	1.00	referent	1.00	referent	1.00	referent
T2	0.88	0.59–1.31	0.79	0.54–1.16	0.84	0.64–1.11
T3	1.14	0.68–1.92	0.67	0.39–1.14	0.90	0.63–1.29
High versus low aggressive						
T1	1.00	referent	1.00	referent	1.00	referent
T2	0.71	0.43–1.16	0.57	0.34–0.93	0.65	0.46–0.92
T3	0.89	0.47–1.70	0.39	0.19–0.82	0.61	0.38–0.97

^a^Adjusted for age, race (in race-unstratified models), total energy intake, education level, smoking status, physical activity, use of nutritional supplements, use of NSAIDs, prostate cancer screening history, family history of prostate cancer, and body mass index.

^b^Tertile ranges and units are as follows.

Refined grain (servings/d): T1 ≤ 3.50, 3.5 < T2 ≤ 5.28, T3 > 5.28.

Whole grain (servings/d): T1 ≤ 0.63, 0.63 < T2 ≤ 1.33, T3 > 1.33.

Total fiber (grams/d): T1 ≤ 16.94, 16.94 < T2 ≤ 24.83, T3 > 24.83.

Insoluble fiber (grams/d): T1 ≤ 10.9, 10.9 < T2 ≤ 16.2, T3 > 16.2.

Soluble fiber (grams/d): T1 ≤ 5.81, 5.81 < T2 ≤ 8.86, T3 > 8.86.
